# Mandatory quality reports in Germany from the hospitals’ point of view: a cross-sectional observational study

**DOI:** 10.1186/1472-6963-12-378

**Published:** 2012-10-31

**Authors:** Silke Auras, Werner de Cruppé, Karl Blum, Max Geraedts

**Affiliations:** 1 Institute for Health Systems Research, Witten/Herdecke University, Alfred-Herrhausen-Str. 50, Witten, 58448, Germany; 2 German Hospital Institute® (DKI®), Hansaallee 201, Duesseldorf, 40549, Germany

**Keywords:** Public reporting, Hospitals, Germany

## Abstract

**Background:**

Public reporting of hospital quality is to enable providers, patients and the public to make comparisons regarding the quality of care and thus contribute to informed decisions. It stimulates quality improvement activities in hospitals and thus positively impacts treatment results. Hospitals often use publicly reported data for further internal or external purposes.

As of 2005, German hospitals are obliged to publish structured quality reports (QR) every two years. This gives them the opportunity to demonstrate their performance by number, type and quality in a transparent way. However, it constitutes a major burden to hospitals to generate and publish data required, and it is yet unknown if hospitals feel adequately represented and at the same time consider the effort appropriate.

This study assesses hospital leaders’ judgement about the capability of QR to put legally defined aims effectively and efficiently into practice. It also explores the additional purposes hospitals use their QR for.

**Methods:**

In a cross-sectional observational study, a representative random sample out of 2,064 German hospitals (N=748) was invited to assess QR via questionnaire; 333 hospitals participated. We recorded the suitability of QR for representing number, type and quality of services, the adequacy of cost and benefits (6-level Likert scales) and additional purposes QR are used for (free text question). For representation purposes, the net sample was weighted for hospital size and hospital ownership (direct standardization). Data was analyzed descriptively and using inferential statistics (chi-2 test) or for the purpose of generating hypotheses.

**Results:**

German hospitals rated the QR as suitable to represent the number of services but less so for the type and quality of services. The cost-benefit ratio was seen as inadequate. There were no significant differences between hospitals of different size or ownership.

Public hospitals additionally used their reports for mostly internal purposes (e.g. comparison with competitors, quality management) whereas private ones used them externally (e.g. communication, marketing) (p=0.024, chi-2 test, hypotheses-generating level).

**Conclusions:**

German hospitals consider the mandatory QR as only partially capable to put the legally defined aims effectively and efficiently into practice. In order for public reporting to achieve its potentially positive effects, the QR must be more closely aligned to the needs of hospitals.

## Background

Public reporting has multiple goals. In the first instance, it is intended to enable patients and referring physicians to make a well-informed choice of healthcare providers by facilitating comparison of the quality of care across providers [1,2]. It is also meant to induce change in the clinical performance of healthcare providers by enhancing quality improvement activities [1,2]. Furthermore, it intends to establish public accountability [2,3]. Despite the widespread practice to publicly report healthcare providers’ performance data, little is known about its actual effects. In 2008, a systematic review by Fung et al. [4] revealed that hospitals’ public reporting is associated with a stimulation of quality improvement activities. Fung et al. could only find inconsistent associations between public reporting and hospital selection or improved effectiveness, respectively. Evidence on the impact of public reporting on patient safety and patient-centeredness is still scant [4].

The quality data collated by hospitals is frequently used for additional purposes. Hospitals routinely distribute their quality data among management, hospital board and medical staff (physicians and nursing staff), one fifth also among ancillary staff (e.g. laboratories) [5]. They use the information to promote collaboration across departments and for internal monitoring of performance [6]. Regarding the internal use of quality data, various effects have been detected; in particular, a stronger integration of best practice guidelines in patient care and improved documentation of treatment processes [5]. Services not being provided before public disclosure have been instituted or planned afterwards [6]. Some hospitals also highlight their outcomes as part of their marketing campaign [2]; some share their quality information with the public, for example via their web pages, internally produced report cards or newsletters [2].

Regarding public reporting in general, numerous positive effects on quality-oriented reorganization have been described, for example heightened attention to quality improvement, new or enhanced quality improvement activities [7,8] and increased investment in hospital staff [5]. Some studies also propose improved clinical outcomes due to activities initiated by public reporting [6,7]. On the other hand, already in 1995 Smith warned of the careless use of performance data [9]. He describes eight examples of unintended behavioural consequences on the part of the internal management of an organization caused by the publication of performance data. These behavioural changes are likely to be dysfunctional [9], i.e. by focussing on phenomena that are quantified and published (“tunnel vision”) and at the same time disregarding the remaining ones [9].

There is little detailed information on the costs of producing and publishing performance data. However, initial costs for the development of measures, analytical methods, and data management systems as well as ongoing costs for data collection, analysis, dissemination, and management of responses have to be considered [6]. Generating physician level quality data is expensive [10] and constitutes a major burden to hospitals [5]. There needs to be a balance between cost, effectiveness, and fairness to providers [6].

Hospitals in Germany are obliged by law to publish structured quality reports (QR) every two years since 2005. Detailed regulations regarding contents, structure and output format of these reports are specified legally. The reports currently contain chapters on structure and performance of the hospital as a whole as well as on each hospital department. Additionally, information on quality assurance and internal quality management is given. For the most part, data on the quality of structures and processes is included, only little information on the quality of outcomes is given. The prescribed data formats are PDF (for publication e.g. on the hospitals’ homepages or in a printed version) and XML/CSV (for the use of data in internet portals which provide hospital comparisons) [11].

However, in the recent version of QR - which was not yet available when data collection for this study began - far more quality data has to be published. Additionally, there are private initiatives on national level (e.g. the “hospital guide” of the TK, a German health insurance, [12]) as well as initiatives on international level (e.g. “Sundhedskvalitet” in Denmark [13]) to better integrate data of the quality of outcomes represented by patient satisfaction data into public reporting.

German QR have three legally defined goals. Firstly, they aim at supporting well-informed choice of hospitals by patients and other interested persons. Secondly, they are meant to guide and support referring physicians as well as sickness funds. And thirdly, German hospitals should be given the opportunity to demonstrate their performance by number, type and quality in a visible and transparent manner [11].

There’s a lack of systematic research on to what extent German hospital QR actually achieve the legally defined aims. Much research has been done on the attitudes and perspectives of patients and referring physicians [14-18] as the most common users of QR. But, to our knowledge, no research has been done on the perspective of hospitals so far. However, it is the hospitals that are burdened the most with collecting and processing data as required and may profit the least from it at the same time - apart from the self-chosen purposes hospitals use QR for as data is already available.

We therefore conducted a survey to assess hospital leaders’ attitude on mandatory quality reporting in Germany by questionnaire. We focused on the hospitals’ opinion regarding the suitability of the QR for meeting the statutory objectives, i.e. the representation of the number, type and quality of services and the cost-benefit ratio of preparing such reports.

## Methods

### Study design

The target population was the entirety of the 2,064 licensed acute care hospitals in Germany which are required by law to produce QR. Using a random sample and a written questionnaire, primary data was collected in 2010. The gross sample of 748 hospitals was representative of the hospital landscape in Germany. In order to enable representative statements based on the net sample, this sample was weighted regarding the distribution of German hospitals in 2009 for two main structural features: the number of beds per hospital and hospital ownership. The number of beds is linked to the number of structural units and therefore the level of specialization of a hospital. Hospital ownership indicates whether a hospital is in public, charitable or private ownership. As a result all results for these two parameters can be considered representative of the German hospital landscape in 2009. However, no conclusions can be made by combining both characteristics, for example about private hospitals with a certain number of beds.

The national distribution of the target population - acute care hospitals in Germany in 2010 - regarding hospital size and hospital ownership can be found in Tables 1 and 2.

**Table 1:** **Target population**: **size of hospitals**, **national distribution in 2010**

**Beds per hospital**	**Number (%) in 2010**
<100 beds	707 (34.3%)
100-299 beds	770 (37.3%)
300-599 beds	428 (20.7%)
≥600 beds	159 (7.7%)
total:	2064 (100%)

Source: The Information System of the Federal Health Monitoring, http://www.gbe-bund.de/.

**Table 2:** **Target population**: **hospital ownership**, **national distribution in 2010**

**Ownership**	**Number (%) in 2010**
public	630 (30.5%)
charitable	755 (36.6%)
private	679 (32.9%)
total:	2064 (100%)

Source: The Information System of the Federal Health Monitoring, http://www.gbe-bund.de/.

The following three questions - from the hospitals’ perspective - were the focus of this study:

Q1: How useful is the QR for demonstrating the number, type and quality of services to the public?

Q2: How appropriate is the cost-benefit ratio?

Q3: For what further purposes do the hospitals use their QR for?

The terms “type” and “quality” were assumed to be known and uniformly understood by the respondents. These terms are defined in the legal specifications of the QR, and we refer to this legal background in the introductory part of our questionnaire.

The bylaws of the local Ethics Committee prescribe ethics approval only for clinical trials, i.e. “biomedical research on human beings” [19]. As our questionnaires didn’t include any biomedical information of the respondent (hospital leaders) or the hospitals’ patients an ethical approval was not required.

### Survey instrument

A questionnaire [see Additional file 1] was used to collect data. The questionnaire was developed by scientists in close collaboration with representatives of the target population - that is leaders of hospital administration - in a consensual manner. The questionnaire consisted of five questions on the following topics, collecting a total of 42 sub-questions:

•Suitability of the QR for demonstrating the number, type and quality of services (Q1, 3 sub-questions)

•Cost-benefit ratio (Q2, 1 sub-question)

•Use of QR for other purposes (Q3, 1 sub-question)

•Detailed assessment of each report section (36 sub-questions)

•Data format for publication (pdf versus xml/csv) (1 sub-question)

Answers were given using 6-level Likert scales (1=very to 6=not at all, 12 sub-questions), dichotomized choice questions (17 sub-questions) and free text questions (13 sub-questions).

The data for research questions Q1 and Q2 were collected using Likert scales where lower numbers indicated better hospital ratings of the respective issue.

The data for research question Q3 came from a free-text question in which multiple responses were allowed.

### Conduct

Data was collected in February 2010 by the German Hospital Institute® (DKI®) which is a leading institution for research, counseling and training in the field of hospital health care. Major associations and institutions of the German hospital industry are members of DKI®. The DKI® regularly assesses hospital leaders’ opinion regarding recent decisions and measures in health care policy. These assessments are always administered in an identical manner being a one-sided questionnaire sent by mail and sent back by fax. Our questionnaire was integrated in such a survey and therefore designed and administered the same way. The faxes were addressed to the administrative hospital leaders. However, it is not known whether hospital leaders internally delegated the completion of the questionnaire. Each questionnaire included an ID identifying the hospital to allow DKI® for connecting recent information gathered with structural hospital data already available at DKI®. Afterwards, data was made anonymous and analyzed at our institute.

### Data analysis

The raw data from the hospitals was weighted using direct standardization with regard to hospital size (number of hospital beds) or hospital ownership in accordance with the actual composition of the German hospital landscape in 2009.

The total of 4 sub-questions with Likert scales (Q1 and Q2) first underwent a weighted descriptive analysis.

Subsequently we used statistical tests to determine whether the responses differed between hospitals of different sizes or ownership. For the statistical testing, the data from the 6-level Likert scales (Q1 and Q2) were dichotomized, i.e. the 3 positive and the 3 negative answer options were grouped together for evaluation. The non-parametric chi-2 test (significance level α = 0.05) was used. To avoid α error inflation by multiple testing, the significance level of 0.05 was divided by the number of tests performed (Bonferroni correction; α(Bonferroni) = 0.05/8 = 0.00625) and the p-values of the tests were compared with α(Bonferroni) = 0.00625.

The free text data (Q3) were initially grouped together under the topics of “internal”, “external” or “internal and external purposes”. Two scientists independently sorted the answers by “being effective internally” or “being effective externally”. Differing classifications were discussed, cleared and consented. Internal purposes were: analysis and comparison with competitors, internal quality management/ accreditation, personal reference, statistics, annual reports, internal information and intranet. In terms of external purposes, data such as outward communication (homepage/ internet, leaflets, brochures, information for referrers), marketing, and recruiting and applicant information was summarized. In each of the weighted samples the data for this question was also tested for differences between hospitals of different sizes or ownership using chi-2. Here, no Bonferroni correction was performed because the grouping of responses (3 groups) and weighting of the data (4 or 3 categories) in some cases resulted in cell ensembles which were too small for reliable statistical testing. Therefore, these results have to be considered hypothesis-generating. The probability that at least one significant result was found by chance alone with a single error probability of α = 0.05 and with two single tests performed is at 1-(1–0.005)^2^ = 0.09756, i.e. just under 10%.

The data was analyzed using PASW Statistics 17.

## Results

### Sample

333 of 748 hospitals participated in the survey (44.5% participation rate).

The description of the sample in terms of the criteria hospital size (number of beds) and hospital ownership can be seen in Tables 3 and 4 both unweighted and standardized for the German federal average in 2009, including the respective weighting factors. The weighting factors show the differences between the gross and the net sample: only few small hospitals and few private hospitals took part in the study (weighting factor > 1) whereas large hospitals and public hospitals participated above-average (weighting factor < 1).

**Table 3:** **Size of hospitals**, **unweighted and weighted for the German federal average in 2009**

**Beds per hospital**	**Number (%) unweighted**	**Number (%) weighted**	**Weighting factor**
<100 beds	52 (15.6%)	104 (31.2%)	1.9964559897
100-299 beds	108 (32.4%)	134 (40.2%)	1.2430830040
300-599 beds	90 (27%)	70 (21%)	0.7750988142
≥600 beds	83 (24.9%)	25 (7.5%)	0.3032822992
total:	333 (100%)	333 (100%)	

**Table 4:** Hospital ownership, unweighted and weighted for the German federal average in 2009

**Ownership**	**Number (%) unweighted**	**Number (%) weighted**	**Weighting factor**
public	152 (45.6%)	106 (31.9%)	0.6995614035
charitable	118 (35.4%)	125 (37.5%)	1.0593220338
private	63 (18.9%)	102 (30.6%)	1.6190476190
total:	333 (100%)	333 (100%)	

### Suitability of the QR to demonstrate the number, type and quality of services in a transparent manner and cost-benefit ratio - description of the 6-level responses

**Figure 1:**
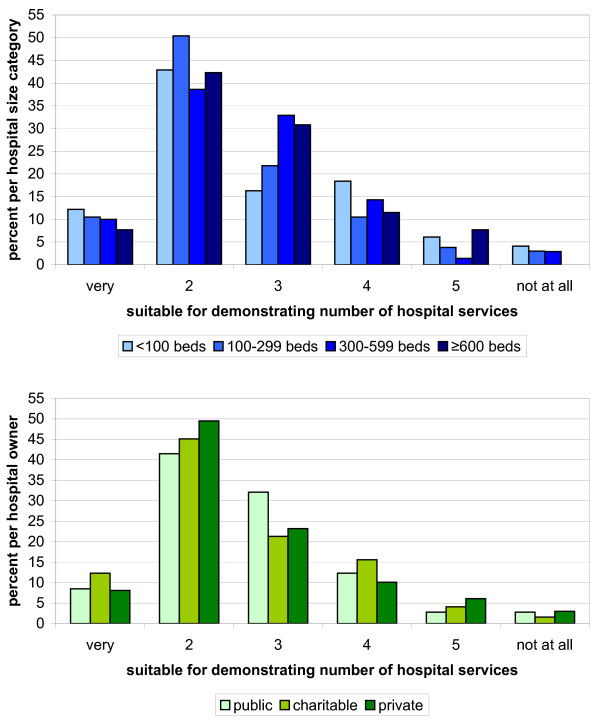
**Hospitals**’ **6**-**level rating of QR suitability for demonstrating the number of services by hospital size (blue) and hospital ownership (green).** The lower the number the better the ratings.

**Figure 2:**
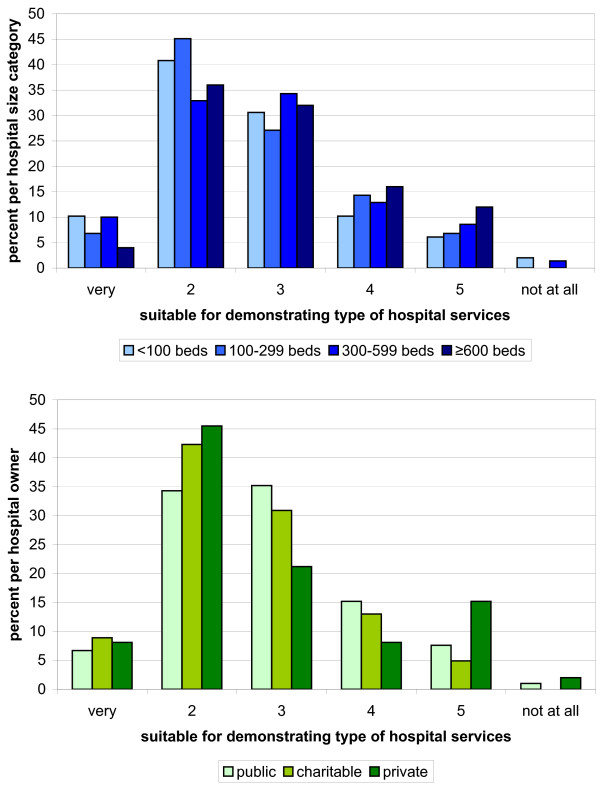
**Hospitals**’ **6**-**level rating of QR suitability for demonstrating the type of services by hospital size (blue) and hospital ownership (green).** The lower the number the better the ratings.

**Figure 3:**
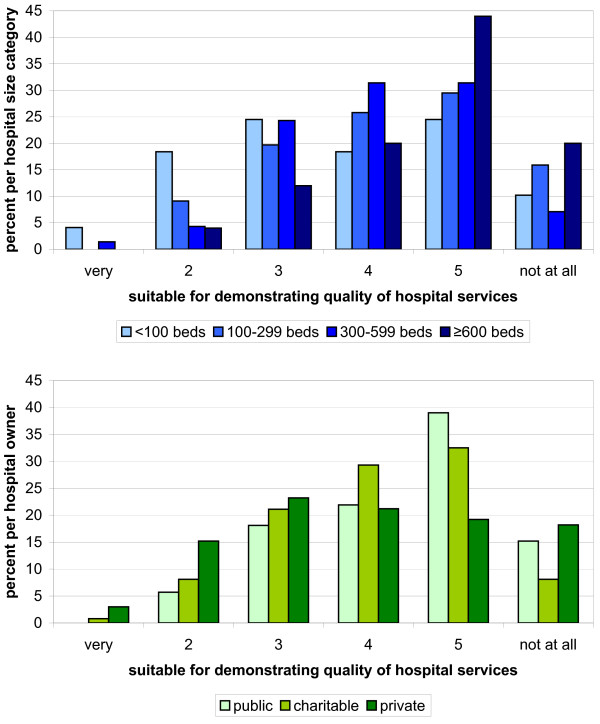
**Hospitals**’ **6**-**level rating of QR suitability for demonstrating the quality of services by hospital size (blue) and hospital ownership (green).** The lower the number the better the ratings.

Research question Q1 investigates the suitability of QR to meet the statutory goals on a 6-level scale from “very suitable” to “not suitable at all”. According to the hospitals in both weighted samples, the QR is suitable for demonstrating the number of services (Figure 1). The QR was considered less suitable or not suitable in terms of showing the type (Figure 2) and quality of services (Figure 3).

**Figure 4:**
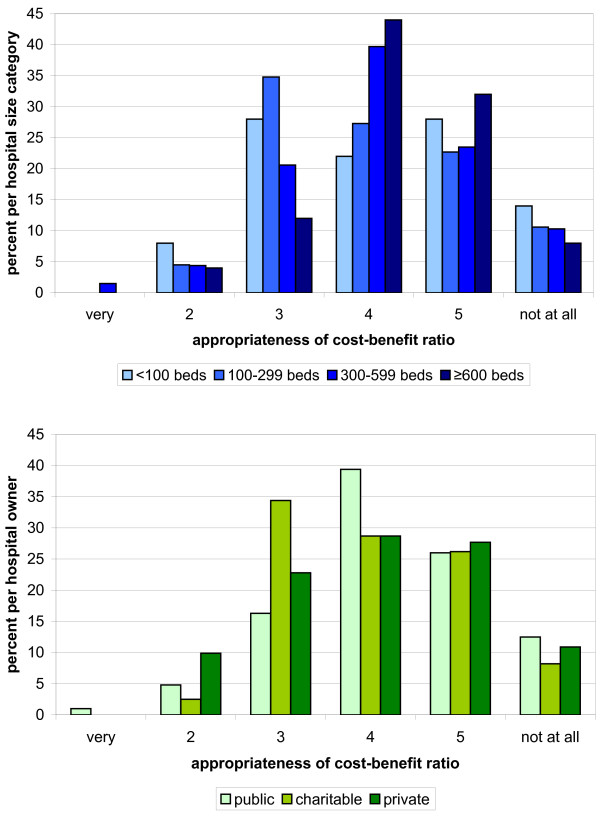
**Hospitals**’ **6**-**level rating of the appropriateness of the cost**-**benefit ratio to generate the QR by hospital size (blue) and hospital ownership (green).** The lower the number the better the ratings.

Research question Q2 examined the cost-benefit ratio of the QR on a 6-level scale from “very appropriate” to “not appropriate at all”. Weighted by number of beds or ownership, at the most 10% of a category rated the cost-benefit ratio with 2 or better (Figure 4).

### Suitability of the QR to demonstrate the number, type and quality of services in a transparent manner and the cost-benefit ratio - statistical testing of the dichotomized responses

After dichotomizing the response categories, the distribution described remained in all four variables across all hospital size categories and ownerships. In particular, hospitals with at least 600 beds and hospitals in public ownership considered the QR as less suitable or not suitable for representing the quality of services (18% and 24% respectively). In these categories, cost-benefit ratio was also more often rated less appropriate or not appropriate (15% and 22% respectively). After Bonferroni correction of the significance level of 0.05 to 0.00625 for research questions Q1 and Q2, the chi-2 test showed no significant difference between hospitals of different sizes or different types of ownership (Tables 5 and 6).

**Table 5:** **Dichotomized responses and results of chi**-**2 tests to assess the suitability of the QR regarding the demonstration of the number**, **type and quality of hospital services and the cost**-**benefit ratio**, **broken down by hospital size**

	<**100 beds** [%]		**100**-**299 beds** [%]		**300**-**599 beds** [%]		≥**600 beds** [%]		**Total** [%]		**p** (**chi**-**2 test**)
	**Rather to very**	**Less to not at all**	**Rather to very**	**Less to not at all**	**Rather to very**	**Less to not at all**	**Rather to very**	**Less to not at all**	**Rather to very**	**Less to not at all**	
Number of services	71.4	28.6	83.0	17.0	82.2	17.8	81.7	18.3	79.2	20.8	0.155
Type of services	81.6	18.4	79.2	20.8	77.4	22.2	74.4	25.6	79.3	20.7	0.852
Quality of services	46.9	53.1	29.2	70.8	30.0	70.0	18.3	81.7	33.9	66.1	0.008
Appropriate cost-benefit ratio	36.0	64.0	39.6	60.4	26.1	73.9	14.8	85.2	33.8	66.2	0.047

**Table 6:** **Dichotomized responses and results of chi**-**2 tests to assess the suitability of the QR regarding the demonstration of the number**, **type and quality of hospital services and the cost**-**benefit ratio**, **broken down by hospital ownership**

	**Public [%]**		**Charitable [%]**		**Private [%]**		**Total [%]**		**p (chi-2 test)**
	**Rather to very**	**Less to not at all**	**Rather to very**	**Less to not at all**	**Rather to very**	**Less to not at all**	**Rather to very**	**Less to not at all**	
Number of services	82.7	17.3	78.4	21.6	80.3	19.7	80.4	19.6	0.727
Type of services	76.0	24.0	81.9	18.1	75.4	24.6	78.0	22.0	0.423
Quality of services	24.0	76.0	30.2	69.8	41.0	59.0	31.5	68.5	0.031
Appropriate cost-benefit ratio	21.6	78.4	37.4	62.6	32.3	67.7	30.8	69.2	0.036

### Use of QR for further internal or external purposes

For further hypothesis-generating evaluation of additional internal and/or external purposes of QR (Q3), 137 hospitals (unweighted) provided legible responses.

With an increasing number of beds per hospital, a greater percentage of hospitals responded (Table 7). At the significance level of 0.05 without Bonferroni correction the p-value of 0.522 (chi-2 test) showed no differences between hospitals of different sizes in terms of further internal and/or external purposes.

**Table 7:** **Responses grouped regarding further purposes of QR**, **broken down by hospital size**

**Number of beds**	**Internal purposes**	**External purposes**	**Internal and external purposes**	**Total**	**Hospitals with legible answers**
<100 beds	14 (41.2%)	10 (29.4%)	10 (29.4%)	34 (100.0%)	34/104 (32.7%)
100-299 beds	26 (51.0%)	14 (27.5%)	11 (21.6%)	51 (100.0%)	51/134 (38.1%)
300-599 beds	12 (37.5%)	15 (46.9%)	5 (15.6%)	32 (100.0%)	32/70 (45.7%)
≥600 beds	5 (41.7%)	5 (41.7%)	2 (16.7%)	12 (100.0%)	12/25 (48.0%)
	57 (44.2%)	44 (34.1%)	28 (21.7%)	129 (100.0%)	129/333 (38.7%)

Public hospitals additionally used their QRs for internal purposes (50%), however, more private ones for external purposes (57%, Table 8) (p = 0.024 chi-2 test).

**Table 8:** **Responses grouped regarding further purposes of QR**, **broken down by hospital ownership**

**Hospital ownership**	**Internal purposes**	**External purposes**	**Internal and external purposes**	**Total**	**Hospitals with legible answers**
public	22 (50.0%)	16 (36.4%)	6 (13.6%)	44 (100.0%)	44/106 (41.5%)
charitable	21 (38.9%)	17 (31.5%)	16 (29.6%)	54 (100.0%)	54/125 (43.2%)
private	13 (35.1%)	21 (56.8%)	3 (8.1%)	37 (100.0%)	37/102 (36.3%)
	56 (41.5%)	54 (40.0%)	25 (18.5%)	135 (100.0%)	135/333 (40.5%)

## Discussion

The majority of German hospitals consider the legally required quality reports (QR) suitable to represent the number of services but less so the type and quality of services. In particular large hospitals (≥ 600 beds) and public hospitals consider the QR as less suitable or not suitable at all to demonstrate the quality of services. These categories also judge the cost-benefit ratio lower or as inappropriate. Significant differences between hospitals of different sizes or different types of ownership do not exist. In terms of additional purposes, public hospitals tend to using QR internally, private hospitals on the other hand for external purposes.

The QR in Germany are supposed to give acute care hospitals as one QR user group the opportunity to demonstrate the number, type and quality of services provided in a transparent manner to the public [11]. For the first time though, our results showed that in their current form, the QR are of limited use for the hospitals, or even barely suitable in this regard. In addition, the majority of respondents indicated that the ratio of benefits of QR and the effort which goes into creating them is not reasonable. Hospitals in Germany therefore, independent of hospital size and ownership, are equally unconvinced and burdened by mandatory quality reporting. This shows that hospitals in Germany as a whole do not think that the QR address their interests adequately.

These results correspond to the assessment of other QR user groups which the legislature intended to target [11]. Other providers, patients and the public also consider the current QR to be of limited use only as an information resource [15,18].

This study showed that hospitals with a minimum of 600 beds are the group in whose opinion the important goal of demonstrating the quality of services has been the least realized while the cost-benefit ratio is rated the most inappropriate. Large hospitals have more specialized departments and usually perform different and more complex procedures than smaller hospitals. Thus there is more chance of failure or negative outcomes, and there is a higher amount of diverse data to deal with. That’s why it is unsurprising that this group gave the worst rating.

Hospitals in public ownership also gave this assessment. In the German hospital landscape, large hospitals are usually in public ownership, which means that these results may well have been caused by a combination of factors. This is supported by the fact that the large hospital categories (300–599 beds, ≥ 600 beds) and the public hospital category are equally overrepresented in the study.

The results of other studies have also been confirmed which showed that in addition to the high production effort [5] many bemoan the high cost involved [6,10] in collecting and processing the quality data. The costs the hospitals incurred in preparing the reports were not collected in this study and should be urgently investigated in another study.

Both in our study and in studies by other authors, some hospitals indicated that they employed the QR data for further internal and external purposes, for example cross-departmental collaboration, internal quality monitoring and various marketing activities [2,5,6]. In our sample, private hospitals which also use the QR for other purposes used them significantly more often for external purposes, for example for PR materials or for marketing. These hospitals work for profit, with some degree of market orientation and competitiveness. Therefore, it seems logical they would use existing data for external representation.

For some of those additional purposes given by the hospitals, occasional positive effects on medical treatment procedures, the documentation thereof and the quality-oriented reorganization of hospitals have been shown [2,5,6], which ultimately could lead to quality of care benefits [6,7]. Whether such effects are also being attained in German hospitals on the basis of the legally required QR must be investigated in further studies.

This study was not carried out using a sample representative of the German hospital landscape but was weighted via direct standardization. The two key structural differences of German hospitals - hospital size (number of beds) and ownership - were only considered individually, not in combination. This means the evaluation is representative only for each single parameter. A random sample of sufficiently large scale could ensure greater generalisability.

## Conclusions

From the hospitals’ point of view the content of the QR must be altered to achieve the desired goals. Hospital leaders don’t feel the quality of services being adequately represented in the reports. Therefore, more quality data should be included. Considering that the requirements of the remaining users are also not met adequately, it should be questioned whether it is justifiable at all to require a single report document for such diverse target groups and target needs.

However, since some positive effects of public reporting are proven, the goal should be to process the appropriate data in each case with the least amount of expenditure and appropriate for the target audience. This way, greater acceptance could be achieved and the potential positive effects of public reporting established and utilized optimally.

## Competing interests

KB is a researcher at the German Hospital Institute® (DKI®) which is a leading institution for research, counseling and training in the field of hospital health care. Major associations and institutions of the German hospital industry are members of DKI®. KB conducted and supervised data collection. He was not involved in data analysis which was - under supervision of WC and MG - exclusively performed by SA at Witten/Herdecke University. The other authors declare that they have no competing interest.

## Authors’ contributions

KB collected data. SA performed data analysis and drafted the manuscript. WC and MG designed the study, supervised data analysis and helped draft the manuscript. KB, WC and MG revised the manuscript critically for important intellectual content. All authors read and approved the final manuscript.

## Pre-publication history

The pre-publication history for this paper can be accessed here:

http://www.biomedcentral.com/1472-6963/12/378/prepub

## Supplementary Material

Additional file 1 **Questionnaire.** This file includes the translated questionnaire used in the survey (original language was German). Click here for file
