# Supplementary material for: Mandatory quality reports in Germany from the hospitals’ point of view: a cross-sectional observational study
Source: BMC Health Serv Res. 2012 Oct 31;12:378. doi: 10.1186/1472-6963-12-378 (PMC3529697; doi:10.1186/1472-6963-12-378)
Supplement: Additional file 1 — Questionnaire. This file includes the translated questionnaire used in the survey (original language was German). [file 1472-6963-12-378-S1.pdf]

universität Witten/Herdecke

Fax: 0211-XX XX XX
Please fax back not later than XX.XX.2010

DEUTSCHES KRANKENHAUS INSTITUT

Hospital Trend regarding the Quality Reports about 2008

According to legal requirements, the mandatory Quality Reports (QR) shall give German hospitals the opportunity to demonstrate their performance by type, number and quality in a visible and transparent manner. The Federal Joint Committee (G-BA)[1] wants to explore if those aims are reached from your perspective and where there is room for improvement.

1 How useful is the information presented in the QR for transparently demonstrating…

	very		-			not at all
a) the types of services provided in your hospital?	①	②	③	④	⑤	⑥
b) the number of services provided in your hospital?	①	②	③	④	⑤	⑥
c) the quality of services provided in your hospital?	①	②	③	④	⑤	⑥

2 How do you judge the single chapters A-D of QR? (Please mark with a cross and explain or rate.)

Chapter A: Data on structure and performance of the hospital*

☐ Important information is missing: → What is missing? ………………………………………..
☐ Unnecessary information is included: → What is dispensable?..
☐ This chapter should be improved: → What should be improved?……...............................
☐ Chapter A is - in its current state - totally all right.

	very high		-			very low
The benefit of Chapter A for our hospital is…	①	②	③	④	⑤	⑥
The costs for Chapter A for our hospital are…	①	②	③	④	⑤	⑥

Chapter B: Data on structure and performance of organisational units/ hospital departments*

☐ Important information is missing: → What is missing? ………………………………………..
☐ Unnecessary information is included: → What is dispensable?..
☐ This chapter should be improved: → What should be improved?……...............................
☐ Chapter B is - in its current state - totally all right.

	very high		-			very low
The benefit of Chapter B for our hospital is…	①	②	③	④	⑤	⑥
The costs for Chapter B for our hospital are…	①	②	③	④	⑤	⑥

Chapter C: Quality Assurance*

☐ Important information is missing: → What is missing? ………………………………………..
☐ Unnecessary information is included: → What is dispensable?..
☐ This chapter should be improved: → What should be improved?……...............................
☐ Chapter C is - in its current state - totally all right.

	very high		-			very low
The benefit of Chapter C for our hospital is…	①	②	③	④	⑤	⑥
The costs for Chapter C for our hospital are…	①	②	③	④	⑤	⑥

Chapter D: Quality Management*

☐ Important information is missing: → What is missing? ………………………………………..
☐ Unnecessary information is included: → What is dispensable?..
☐ This chapter should be improved: → What should be improved?……...............................
☐ Chapter D is - in its current state - totally all right.

	very high		-			very low
The benefit of Chapter D for our hospital is…	①	②	③	④	⑤	⑥
The costs for Chapter D for our hospital are…	①	②	③	④	⑤	⑥

3 For what other purposes do you use the QR? (please explain)

……………………………………………………………………………………………………

4 How appropriate do you think is the cost-benefit-ratio - also considering your individual other purposes?

very		-			not at all
①	②	③	④	⑤	⑥

5 Which of the publication formats do you think is more suitable to reach the above-named "goals of transparency"?

☐ pdf-Version ☐ XML/CSV-Version (for the use of data in internet portals)

*Please feel free to add further comments on a separate page.

[1] The G-BA is the highest decision-making body of the joint self-government of physicians, dentists, hospitals and health insurance funds in Germany.
